# Probiotics for Alleviating Alcoholic Liver Injury

**DOI:** 10.1155/2019/9097276

**Published:** 2019-05-27

**Authors:** Zelin Gu, Yanlong Liu, Shumeng Hu, Ying You, Jiaqi Wen, Wancong Li, Yuhua Wang

**Affiliations:** ^1^College of Food Science and Engineering, Jilin Agricultural University, Changchun, China; ^2^College of Pharmaceutical Sciences, Wenzhou Medical University, Wenzhou, China; ^3^National Processing Laboratory for Soybean Industry and Technology, Changchun, China; ^4^National Engineering Laboratory for Wheat and Corn Deep Processing, Changchun, China

## Abstract

Many animal experiments and clinical trials showed that probiotics are effective for the treatment of alcoholic liver disease. Alcohol disrupts the composition of intestinal flora; probiotics modulate the gut microbiota and reverse alcohol-associated intestinal barrier dysfunction by decreasing intestinal mucosal permeability and preventing intestinal bacteria from translocating. Probiotics enhance immune responses and reduce the levels of alcohol-induced inflammatory cytokines and reactive oxygen species (ROS) production in the liver and intestine. Probiotics also increase fatty acid *β*-oxidation and reduce lipogenesis, combating alcohol-induced hepatic steatosis. In this review, we summarize the current knowledge regarding the mechanism of action of probiotics for reducing the effects of alcoholic liver disease.

## 1. Introduction

With economic development and increased levels of social activity, alcohol consumption is increasing worldwide. An increasing number of patients suffer from alcoholic liver disease. According to statistics, 80% of drinkers exhibit characteristics of alcoholic liver injury, of which 10%–35% progress to alcoholic hepatitis and 10%–20% may develop alcoholic cirrhosis [1]. Alcohol abstinence can reverse mild alcoholic liver injury but not the irreversible injury of cirrhosis. To date, the details of alcoholic liver damage pathogenesis remain unclear, and effective drugs to treat the condition have not been discovered. Probiotics are functional products; their beneficial physiological and biochemical functions have been demonstrated in humans. Studies have demonstrated that probiotics effectively attenuate alcoholic liver disease as well as improve liver function [2–4]. In this paper, we summarize the effects of probiotics regarding improvement of alcoholic liver disease.

## 2. Alcoholic Liver Injury

Alcoholic liver disease (ALD) is a major of chronic liver disease, caused by excessive alcohol ingestion. In the initial stages, alcohol consumption induces development of fatty liver, gradually developing into hepatitis, liver fibrosis, and cirrhosis and often deteriorating to liver cancer [5–7]. Although abstinence can relieve alcoholic liver injury, it cannot completely reverse excessive alcohol consumption-induced liver injury. Treatments for alcoholic liver injury are few in number; therefore, it is urgent to find an effective method for the treatment of alcoholic liver injury. Alcohol exposure compromises intestinal barrier function, leading to increased intestinal permeability and accumulation of endotoxin in the blood, causing liver damage [8, 9]. Long-term or heavy drinking disrupts liver function, via mitochondrial injury and hepatic accumulation of acetaldehyde. Acetaldehyde combines with several proteins in the liver and causes functional protein denaturation and antigen exposure that stimulates the immune system to produce antibodies, causing liver damage [10, 11]. In recent years, the mechanisms of action of probiotics for alleviating alcoholic liver injury have been a subject of study.

## 3. Probiotics

Probiotics are living microorganisms that play beneficial roles, depending on the dose [12], directly maintaining the balance of intestinal microbiota [13]. The physiological functions of probiotics are achieved directly or indirectly by adjusting the composition of the host intestinal microbiota, activating the endogenous microbial community and regulating the immune system [14]. Oral probiotics can cure or alleviate a variety of gastrointestinal diseases. Some studies have confirmed that oral probiotics effectively alleviate lactose intolerance; prevent gastroenteritis, constipation, and diarrhea; and modulate the gut microbiota [15, 16]. Kirpich was the earliest to use probiotics to treat ALD patients, showing that probiotics (*B. bifidum* and *L. plantarum* 8PA3) significantly increased the number of *Lactobacillus* and *Bifidobacterium* in human feces and significantly altered the serum levels of ALT, low-density lipoprotein (LDL), and total bilirubin (STB) [4]. Therefore, many investigators began to pay attention on the role of probiotics in alcoholic liver disease. They used a variety of probiotic strains, most often *Lactobacillus rhamnosus* GG (LGG). LGG is a short Gram-positive heterofermentative facultative anaerobe which was isolated in 1983. LGG has been shown to be effective in the treatment of ALD.

## 4. Mechanisms of Probiotics in Improvement of Alcoholic Liver Injury

### 4.1. Probiotics and Antioxidant Activity

**Figure 1:**
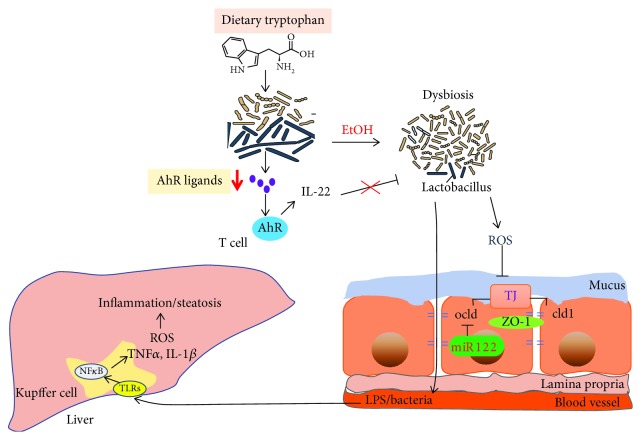
The effect of alcohol on the gut-liver axis. Alcohol significantly changes intestinal microbiota diversity, reduces intestinal epithelial tight junction protein expression, and increases intestinal mucosal permeability, leading to barrier dysfunction and endotoxin translocated into the blood, inducing inflammatory cytokine and ROS production in the intestine and liver, causing hepatic steatosis and inflammation. Alcohol disorders the gut microbiota and decreases AhR ligand and IL-22 production. Alcohol exposure increased intestinal miR122a expression, decreasing occludin expression.

Reactive oxygen species (ROS) are highly reactive oxygen-containing molecules, ions, or groups that interact with one another and damage cellular molecule complexes, especially in the liver. In vivo, ROS causes oxidation of unsaturated fatty acids to produce lipid peroxides that induce fatty acid side-chain reactions, creating lipid hydroperoxides and malondialdehyde (MDA), which lead to damage of biological macromolecules (including protein and DNA), thereby affecting physiological metabolic activity and impairing cell structure and function [17–19]. ROS negatively regulate these mechanisms in alcoholic liver disease [20, 21]. Alcohol consumption induced the overproduction of ROS and inhibited fatty acid oxidation in the liver [22, 23], leading to ROS-mediated liver injury [24, 25] (Figure 1).

**Figure 2:**
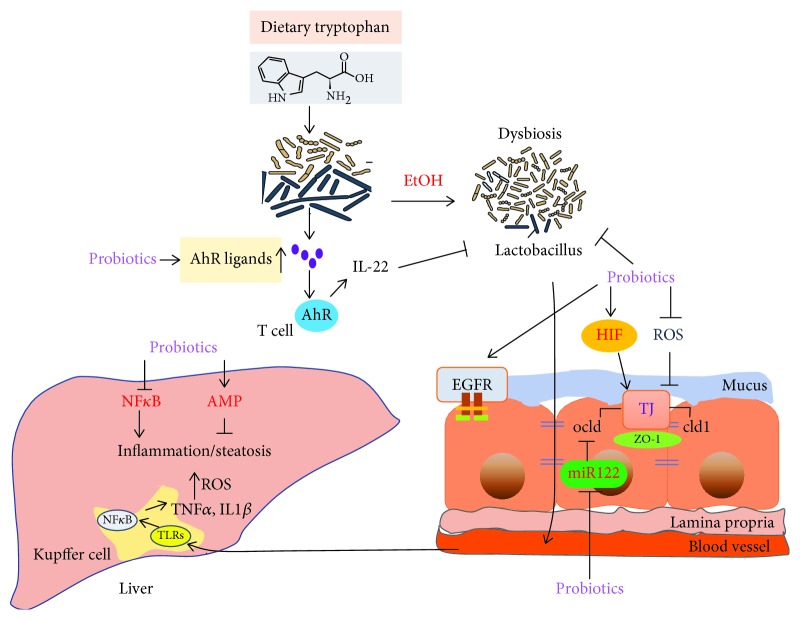
Probiotics function in gut-liver mechanisms. Probiotics and related products prevent ethanol-induced effects in the intestine and the liver via multiple mechanisms: (1) enhancement of antioxidant activity; (2) reduction of inflammatory cytokine expression; (3) hepatic AMPK and induction of lipid metabolism; (4) enhancement of intestinal tight junction ZO-1, claudin-1, and occludin expression via increased intestinal HIF signaling; (5) inhibition of miR122a expression leading to occludin upregulation; (6) activation of EGFR and preservation of barrier function in intestinal epithelial cells; and (7) positive modification of gut microbiota and increase AhR ligands.

Studies demonstrated that probiotics enhance antioxidant activity to alleviate alcohol-induced liver injury (Figure 2). LGG treatment relieved alcohol-induced ROS accumulation in the ileum and Caco-2 cells [26]. LGG culture supernatant (LGG-s) pretreatment protected hepatic and ileal cells from oxidative stress and suppressed ROS formation [27]. Wang et al. showed that *L. rhamnosus B10* significantly decreased the levels of ALT, LPS, and MDA; increased the activity of superoxide dismutase; and relieved alcohol-induced fatty degeneration and oxidative injury [28]. In the liver, ROS increased hepatic CYP2E1 protein and mRNA expression [24, 29] and substantially decreased Nrf-2 protein expression, all of which was ameliorated by LGG treatment [30]. These results suggested that probiotics reduce oxidative stress and promote the production of antioxidants to alleviate oxidative damage in the liver.

### 4.2. Probiotics Improve Alcohol-Induced Lipid Metabolism

The AMP-activated protein kinase (AMPK) signaling pathway is an important energy metabolic pathway characterized by a series of cascade reactions that activate catabolism and deactivate anabolism [31]. AMPK regulates lipid metabolism by manipulation of several transcription factors, including peroxisome proliferator-activated receptor-*α* (PPAR*α*) and sterol regulatory element-binding protein 1 (SREBP-1), which play important roles in lipogenesis and fatty acid oxidation [32, 33]. PPAR*α* dominates the expression of lipid oxidation genes, including carnitine palmitoyltransferase-1 (CPT-1), acyl-CoA oxidase (ACO), acyl-CoA synthetase long-chain 1 (ACSL-1), and others. SREBP-1 regulates lipid synthesis by mediating its downstream genes, including acetyl-CoA carboxylase *α* (ACC*α*), fatty acid synthase (FAS), and stearoyl CoA desaturase 1 (SCD-1). Studies have shown that increased AMPK phosphorylation is effective in the treatment of alcoholic fatty liver [34, 35].

Chronic or acute alcohol consumption decreased AMPK and acetyl-CoA carboxylase (ACC) phosphorylation and increased malonyl-Co-A (MCA) production, leading to abnormal lipid metabolism in the liver [33, 36]. A study showed that LGG-s activated AMPK and suppressed alcohol-induced lipid accumulation via reduced expression of SREBP-1 and upregulated levels of CPT-1 and PPAR*α* [37] (Figure 2). Zhang et al. observed that LGG-s stimulated adiponectin secretion and improved insulin sensitivity. LGG-s supplementation significantly increased the expression of Bcl-2 and inhibited the expression of Bax, suggesting that LGG-s had an important effect on protection against alcohol-induced hepatocyte apoptosis [37]. In short, LGG-s increases fatty acid oxidation to prevent chronic alcohol-induced liver steatosis and injury and decreases lipogenesis and hepatocyte apoptosis.

### 4.3. Probiotics Reduce Inflammatory Cytokine Expression in the Liver and Intestine

Alcohol-induced barrier dysfunction results from local and systemic production of proinflammatory cytokines such as TNF-*α* and IL-1*β* [38–40] (Figure 1). Once the gut barrier function is compromised, there is translocation of bacteria and bacterial products released into the blood; subsequently, a large number of Kupffer cells accumulate and toll-like receptors (TLRs) on the surface of liver Kupffer cells combine with endotoxin to activate mitogen-activated protein kinase (MAPK) and nuclear factor *κ*B (NF-*κ*B), producing inflammatory cytokines, including TNF-*α* and interleukin (IL-6, IL-1*β*) [16, 40, 41]. Our group demonstrated that LGG-s treatment decreased alcohol-induced inflammatory cytokine (TNF-a) production via inhibition of TLR4- and TLR5-mediated endotoxin activation [30] (Figure 2). Bajaj et al. showed that probiotic administration significantly decreased the amount of endotoxin and TNF-*α* in patients with mild hepatic encephalopathy [42].

NF-*κ*B also is activated in intestinal TLRs, important promoters of intestinal immunity [43–45]; probiotics act on the immune system through TLRs. In 2009, Miyauchi et al. showed that LGG reduced the secretion levels of TNF-*α* from the intestinal mucosa and restored the integrity and barrier function of epithelial cells [46]. Probiotic supplementation decreased TNF-a and TLR (TLR4, TLR5) expression in the intestine and decreased the phosphorylation of p38 MAP kinase in mice with ALD [30, 40]. LGG-fermented milk contains two soluble proteins, p40 and p75, both of which have been reported to promote survival and growth of intestinal epithelial cells through activation of the epidermal growth factor receptor (EGFR). EGFR and Akt activation prevented cytokine-induced inflammation and intestinal epithelial cell apoptosis [47] (Figure 2).

### 4.4. Probiotics Enhance Intestinal Barrier Function and Modulate the Mucosal Immune System

In 2006, Ewaschuk and Dieleman showed that LGG directly or indirectly affected intestinal epithelial cells, strengthening intestinal mucosal barrier function and regulating the immune system [48] (Figure 2). Probiotics significantly increase the content of short-chain fatty acids (SCFA) that provide the energy metabolic substrate in the intestine and promote absorption of sodium ions, proliferation of colon cells, and intestinal mucous growth [49, 50]. A recent study found that the probiotic *Akkermansia muciniphila* promoted intestinal barrier integrity via enhancing the expression of tight junction (TJ) proteins claudin-3 and occludin, thereby ameliorating alcohol liver injury [51].

Alcohol consumption decreased the expression of the hypoxia-inducible factor (HIF). HIF is a master transcription factor involved in maintaining barrier function. It increases the expression of the intestinal trefoil factor (ITF), xenobiotic clearance by P-glycoprotein (P-gp), and various other nucleotide signaling pathways [52]. Wang et al. used a C57BL/6N mouse model to show that chronic alcohol treatment significantly decreased the expression levels of ITF and TJ proteins claudin-1, ZO-1, and occludin, while LGG treatment significantly improved these conditions [26]. HIF-1*α* regulated a variety of genes in the intestine, including antimicrobial peptides, *β*-defensin 1, and CRAMP [53–55]. LGG and LGG-s increased HIF expression in chronic and acute alcohol liver diseases in mice; we also demonstrated that knockdown of HIF1/2*α* resulted in increased permeability and decreased trans-epithelial electrical resistance in Caco-2 cells [26, 27]. Alcohol (EtOh) impaired epithelial integrity, and probiotic preserved barrier function by maintaining HIF activity and mucus molecules.

Probiotics also prevent and mitigate alcohol-induced disruption of colonic epithelial tight junctions, endotoxemia, and liver damage by an epidermal growth factor receptor- (EGFR-) dependent mechanism [56]. EGF receptor transactivation, prevented apoptosis, and preserved barrier function in intestinal epithelial cells [57].

There are many mechanisms of probiotics that regulate tight junction proteins. MicroRNA 122a (miR122a) is a target of tight junction occludin [58]. Alcohol induced the overexpression of miR122a in the intestine, decreasing occludin expression (Figure 1). Probiotic LGG-s suppressed miR122a expression and restored intestinal occludin protein expression in ALD mice [59] (Figure 2).

### 4.5. Probiotics Regulate Intestinal Flora

There is a close relationship between gut microbiota and human health. They are crucial actors in the pathogenesis of liver disease [26, 60, 61]. Many of these effects are mediated by metabolites that are produced by microbes. Tryptophan is an amino acid that is metabolized into aryl hydrocarbon receptor (AhR) ligands by intestinal microbiota in mice and humans [62]. AhR activation-mediated intestinal T cells secrete IL-22 that modulates the gut microbiota [63, 64]. Both chronic and acute alcohol consumption leads to intestinal dysbiosis and pathogenic bacterial overgrowth [24, 65–68] and decreased AhR ligand production [69]. Disturbance of gut microbiota increased the intestinal permeability and decreased TJ protein expression [63] (Figure 1).

One of the major mechanisms of probiotic function is alteration of gut microbiota. Gut microbiota play a key role in the immune system; imbalance of the intestinal microbiota stimulates the immune system, promoting chronic liver inflammation [8, 9]. *Bifidobacteria* and *Lactobacillus* are the predominant genera when there is a significant difference between the host and individual bacterial species [70, 71], which prevents the growth of anaerobic Gram-positive bacteria, inhibits the growth of Gram-negative bacteria, enhances phagocytic activity, and promotes the secretion of IgA, thereby enhancing cellular immune function [72]. Supplementation with probiotics naturally produced AhR ligands, promoting IL-22 production; intestinal regenerating islet-derived 3 gamma (Reg3g) expression was mediated by IL-22 which improved alcohol-induced liver injury and prevented microbiota disorder [69] (Figure 2).

Mutlu and colleagues used a rat model with ALD and found that microbiota composition in the colon did not change with 4–6-week alcohol consumption until the 10th week, and this was prevented by probiotic treatment [73]. In 1984, Bode was the first to study changes in gut microbiota in patients with alcoholic liver disease. They found that, compared with the normal control group, the number of Gram-negative anaerobic bacteria was significantly greater, and the same situation was observed for aerobic bacteria. This conclusion was helpful in the analysis of the function of the small intestine in patients with alcoholic liver [74]. Alcohol consumption decreased the number of *Bacteroides* and *Firmicutes* and increased the number of *Proteobacteria* and *Actinobacteria* in the intestine [75]. Alcohol induced the greatest expansion of *Corynebacterium* and *Alcaligenes*; the proliferation of *Alcaligenes* increased intestinal pH and led to pathogenic bacteria overgrowth. The addition of LGG suppressed the overgrowth of Gram-negative bacteria *Alcaligenes* and Gram-positive bacteria *Corynebacterium* [75].

*Akkermansia muciniphila* constitutes 1% to 4% of fecal microbiota in healthy individuals [76, 77]. *A. muciniphila* is a Gram-negative anaerobic commensal that utilizes host-derived mucins such as carbon and nitrogen [78], producing SCFA propionates. SCFA propionates act as histone deacetylase inhibitors, suggesting that they can epigenetically influence host gene expression [79]. Alcohol exposure diminished intestinal *A. muciniphila* abundance in both mice and humans, and *A. muciniphila* supplementation reversed alcohol-mediated liver injury and gut microbiota dysbiosis [51].

## 5. Future Perspectives of Microbial-Associated Therapy in ALD

Alcohol-induced liver disease significantly changed the gut microbial composition. In preclinical models, investigators found that fecal microbiota transfer caused the transmissibility of alcohol-related liver disease [80, 81]. Modulation of gut microbial composition and fecal microbiota transplantation (FMT) from healthy individuals will be two of the logical treatments in the future. Philips et al. showed daily FMT from several donors for 7 days improved liver function and reduced potentially pathogenic species [82]. Clinical trials found that FMT was beneficial in severe alcoholic hepatitis, hepatic encephalopathy, nonalcoholic fatty liver disease, hepatitis B-related chronic liver disease, and primary sclerosing cholangitis [83].

Restoration of microbial metabolites will be another logical treatment approach in the future. Supplementation with probiotics increases AhR ligand, SCFA, and LCFA production by modulating the gut microbiota; bioengineered bacteria can deliver therapeutics to the microbiota or host. Alternatively, drugs can be used to change and modulate bacterial enzymes or pathways [83].

Although we have some understanding of the interactions between the intestinal microbiome and the host, we lack a comprehensive understanding of the function of the intestinal microbiome in alcoholic liver disease. Larger and long-term clinical trials on the modification of intestinal microorganisms are required.

## 6. Conclusion

A large number of studies have found and confirmed that probiotics have the effect on alleviating alcoholic liver injury. Probiotics increase TJ protein expression and prevent disturbances of the intestinal barrier function in ALD. Intestinal flora is closely related to human health. Probiotics modulate the gut microbiota and immune responses, decreasing inflammatory cytokine and ROS expression in the intestine and liver. Probiotics also reduce fat accumulation in the liver by increasing fatty acid *β*-oxidation.

## Abbreviations

ALD: Alcoholic liver disease
LGG: L. rhamnosus GG
LGG-s: L. rhamnosus GG culture supernatant
HIF: Hypoxia-inducible factor
ITF: Intestinal trefoil factor
P-gp: P-glycoprotein
TJ: Tight junction
CRAMP: Cathelin-related antimicrobial peptide
TNF-*α*: Tumor necrosis factor-*α*
ROS: Reactive oxygen species
MAPK: Mitogen-activated protein kinase
TLRs: Toll-like receptors
NF-*κ*B: Nuclear factor *κ*B
AMPK: Adenosine monophosphate-activated protein kinase
EGFR: Epidermal growth factor receptor
AhR: Aryl hydrocarbon receptor
Reg3g: Regenerating islet-derived 3 gamma.
